# Transplantation, gene therapy and intestinal pathology in MNGIE patients and mice

**DOI:** 10.1186/s12876-018-0881-0

**Published:** 2018-10-19

**Authors:** Rana Yadak, Max V. Boot, Niek P. van Til, Dominique Cazals-Hatem, Armin Finkenstedt, Elly Bogaerts, Irenaeus F. de Coo, Marianna Bugiani

**Affiliations:** 1 Department of Neurology, Erasmus University Medical Center, Rotterdam, The Netherlands; 2 Department of Hematology, Erasmus University Medical Center, Rotterdam, The Netherlands; 3 Department of Pathology, VU University Medical Center, Amsterdam, The Netherlands; 4 Laboratory of Translational Immunology, University Medical Center Utrecht, Utrecht, The Netherlands; 5 Department of Pathology, Beaujon Hospital, Clichy, France; 6 Department of Medicine I, Medical University of Innsbruck, Innsbruck, Austria; 7 Department of Clinical Genetics, Erasmus University Medical Center, Rotterdam, The Netherlands; 8 Department of Clinical Genetics, Maastricht University Medical Center, Maastricht, The Netherlands

**Keywords:** Mitochondrial neurogastrointestinal encephalomyopathy, Gastrointestinal symptoms, HSCT, HCSGT, Hematopoietic stem cell gene therapy, Cajal cells, CD117/c-kit

## Abstract

**Background:**

Gastrointestinal complications are the main cause of death in patients with mitochondrial neurogastrointestinal encephalomyopathy (MNGIE). Available treatments often restore biochemical homeostasis, but fail to cure gastrointestinal symptoms.

**Methods:**

We evaluated the small intestine neuromuscular pathology of an untreated MNGIE patient and two recipients of hematopoietic stem cells, focusing on enteric neurons and glia. Additionally, we evaluated the intestinal neuromuscular pathology in a mouse model of MNGIE treated with hematopoietic stem cell gene therapy. Quantification of muscle wall thickness and ganglion cell density was performed blind to the genotype with ImageJ. Significance of differences between groups was determined by two-tailed Mann-Whitney *U* test (*P* < 0.05).

**Results:**

Our data confirm that MNGIE presents with muscle atrophy and loss of Cajal cells and CD117/c-kit immunoreactivity in the small intestine. We also show that hematopoietic stem cell transplantation does not benefit human intestinal pathology at least on short-term.

**Conclusions:**

We suggest that hematopoietic stem cell transplantation may be insufficient to restore intestinal neuropathology, especially at later stages of MNGIE. As interstitial Cajal cells and their networks play a key role in development of gastrointestinal dysmotility, alternative therapeutic approaches taking absence of these cells into account could be required.

## Background

Mitochondrial neurogastrointestinal encephalomyopathy (MNGIE) is a rare inherited metabolic disorder caused by loss-of-function mutations in the nuclear gene *TYMP* leading to thymidine (Thd) and deoxyuridine (d-Urd) accumulation [1]. Alongside classic neurological signs (external ophtalmoplegia, leukoencephalopathy and sensorimotor peripheral neuropathy), chronic intestinal pseudo-obstruction (CIPO) is reported in almost all MNGIE patients and occurs at onset in 45–67% of cases [2, 3]. Other gastrointestinal symptoms include early satiety, nausea, dysphagia, post-prandial emesis, abdominal pain and/or distention and diarrhea [4].

Allogeneic hematopoietic stem cell transplantation (HSCT) corrects the biochemical metabolic imbalance as donor-derived leucocytes and platelets are rich in thymidine phosphorylase [4]. It is effective to relieve CIPO in few reported MNGIE cases [5] although malnutrition often persists and most cases rely on nutritional support [6]. Neurogenic and myogenic changes and alterations of the interstitial Cajal cells, the gut pacemakers, were reported in MNGIE patients [7–10]. Restoration of gastrointestinal integrity by available treatments however has not yet been addressed. Also, whether small intestine pathology is recapitulated in the mouse model of MNGIE is not known [11, 12]. In this study, we also evaluated the effects of treatment on small intestinal pathology of MNGIE patients and mice.

## Methods

### MNGIE patients and controls

Table 1 reports the demographic data of three MNGIE patients and three controls. Patients were diagnosed based on clinical, biochemical, and molecular features [1]. One patient was untreated, two received HSCT. Written informed consent was obtained for all subjects. Human control tissue was obtained from surgical resections and employed according to the Dutch law where they can be used for secondary use when no objection has been received. This is also valid abroad, because the country of origin is determinant for the rules and regulations for secondary use.

**Table 1:** Clinical and molecular data of MNGIE patients and controls

Patient	MNGIE-1	MNGIE-2[22]	MNGIE-3	Control-1	Control-2	Control-3
Age of onset	18 y	23 y	10 y	NA	NA	NA
GI symptoms	Diarrhea, vomiting, weight loss, abdominal pain, liver steatosis	Diarrhea, weight loss, liver steatosis	Diarrhea, weight loss, abdominal pain	NA	NA	NA
Extra-GI symptoms	Ptosis, peripheral neuropathy, neurogenic bladder, leukoencephalopathy, lactic acidosis, hypertriglyceridemia	External ophtalmoplegia, peripheral neuropathy, leukoencephalopathy	Retinopathy, peripheral neuropathy, leukoencephalopathy	NA	NA	NA
Diagnosis	TP deficiency	Urinary d-Urd, c.866A > C in *TYMP*	c.866A > C in *TYMP*	Pancreatitis	IOPN	GIST
Treatment of MNGIE (age)	None	Allogeneic HSCT (34 y)	Allogeneic HSCT (17 y)	–	–	–
Follow-up	Alive (6 y)	Multi-organ failure; died 18 days after treatment	GVHD, sepsis; died 6 months after treatment	NA	NA	NA

*GI* gastrointestinal. *NA* not available, *TP* thymidine phosphorylase enzyme, *d-Urd* deoxyuridine, *TYMP* thymidine phosphorylase *IOPN* intra-ductal oncocytic papillary neoplasm, *GIST* gastrointestinal stromal tumor, *HSCT* hematopoietic stem cell transplantation, *GVHD* graft-versus-host disease

### MNGIE mice

*Tymp*^−/-^*Upp1*^−/−^ (KO) and *Tymp*^+/+^*Upp1*^+/+^ wild type (WT) mice [11] were bred in filter top cages and fed ad libitum with autoclaved water and irradiated chow. During the course of experiments, mice were monitored carefully for any signs of discomfort. Animal experiments were approved by the ethical committee of the Erasmus University Medical Center, Rotterdam, in accordance with Dutch legislation. pRRL.PGK.TYMP.bPRE4*.SIN self-inactivating lentiviral transfer plasmid containing the human PGK promoter driving *TYMP* sequence [13], and the third-generation [14] packaging and envelop plasmids were used to generate LV-PGK-TP vector particles, by calcium-phosphate precipitation of HEK293T cells [15]. Titration was performed on HeLa cells and titers determined by quantitative polymerase chain reaction (qPCR). Donor KO male bone marrow lineage-depleted (Lin-) cells (BD Biosciences) were transduced overnight with the lentiviral vector at a multiplicity of infection 10 in serum-free modified Dulbecco’s medium with supplements, [16] conditioned with murine stem cell factor, human Flt3-L, and murine thrombopoietin. Five to 10-week-old recipient KO female mice received 6Gy total body irradiation 24 h prior to tail vein injection of 0.5 × 10^6^ Lin-transduced cells. The experiments included two untreated control groups; *Tymp*^−/-^*Upp1*^−/−^ (KO) and *Tymp*^+/+^*Upp1*^+/+^ wild type (WT) mice and one *Tymp*^−/-^*Upp1*^−/−^ treatment group (PGK-TP). *Tymp*^−/-^*Upp1*^−/−^ mice were randomly allocated to become either a control untreated mice (KO) or to receive the treatment (PGK-TP). The primary experimental outcomes assessed include: nucleoside levels in urine samples, molecular chimerism and vector copy per cell, and pathological evaluation of the intestine.

Bone marrow genomic DNA (Bioké, Leiden, The Netherlands) was used as template for qPCR using primers and SYBR Green PCR master mix (Applied Biosystems, Foster City, CA; Eurogentec, Maastricht, The Netherlands). PCR reactions were carried out in the ABI7900, Taqman machine, and analysis performed with SDS2.2.2 software (Applied Biosystems). The Cycle threshold values were compared against a standard curve obtained from mouse 3T3 to calculate average vector copy per cell, or from male mice bone marrow to calculate Y chromosome chimerism.

High performance liquid chromatography (Shimadzu, LC20 series with a binary pump and Photodiode array detector) [17] equipped with an Alltima C18 5 μ, 250 mm × 4.6 mm column and Alltima C18 5 μ guard column was used to measure urinary Thd and d-Urd.

### Pathological analysis

Mice were euthanized by inhalation of a 5% CO_2_ / 95% O_2_ followed by 100% CO_2_ for 4 min, and transcardially perfused with PBS to remove blood. Human and mouse formalin-fixed paraffin-embedded 5-μm-thick small intestine tissue sections were routinely stained for Hematoxylin-Eosin and Phosphotungstic acid-hematoxylin and immunostained as described [18] against smooth muscle actin (SMA, Dako, 1:200), CD117/c-kit (Dako, 1:50), calretinin (Dako, 1:200), NeuN (Millipore, 1:100), CD3 (Dako, 1:250), CD8 (Dako, 1:50) and glial fibrillary acidic protein (GFAP, Dako, 1:300). Immunoreactivity was detected using 3,3'-Diaminobenzidin or Liquid Permanent Red as chromogen. Pictures were taken with a Leica DM3000 microscope. Muscle thickness was measured on transversally cut intestinal sections (*N* = 40 for mice, *N* ≥ 25 for humans). Quantification of muscle wall thickness and ganglion cell density was performed blind to the genotype with ImageJ.

### Statistical analysis

Data were analyzed with Graph Pad-Prism5 (version 5.03). Significance of differences between groups was determined by two-tailed Mann-Whitney *U* test (*P* < 0.05).

## Results

### Small intestine pathology in MNGIE patients

**Fig. 1:**
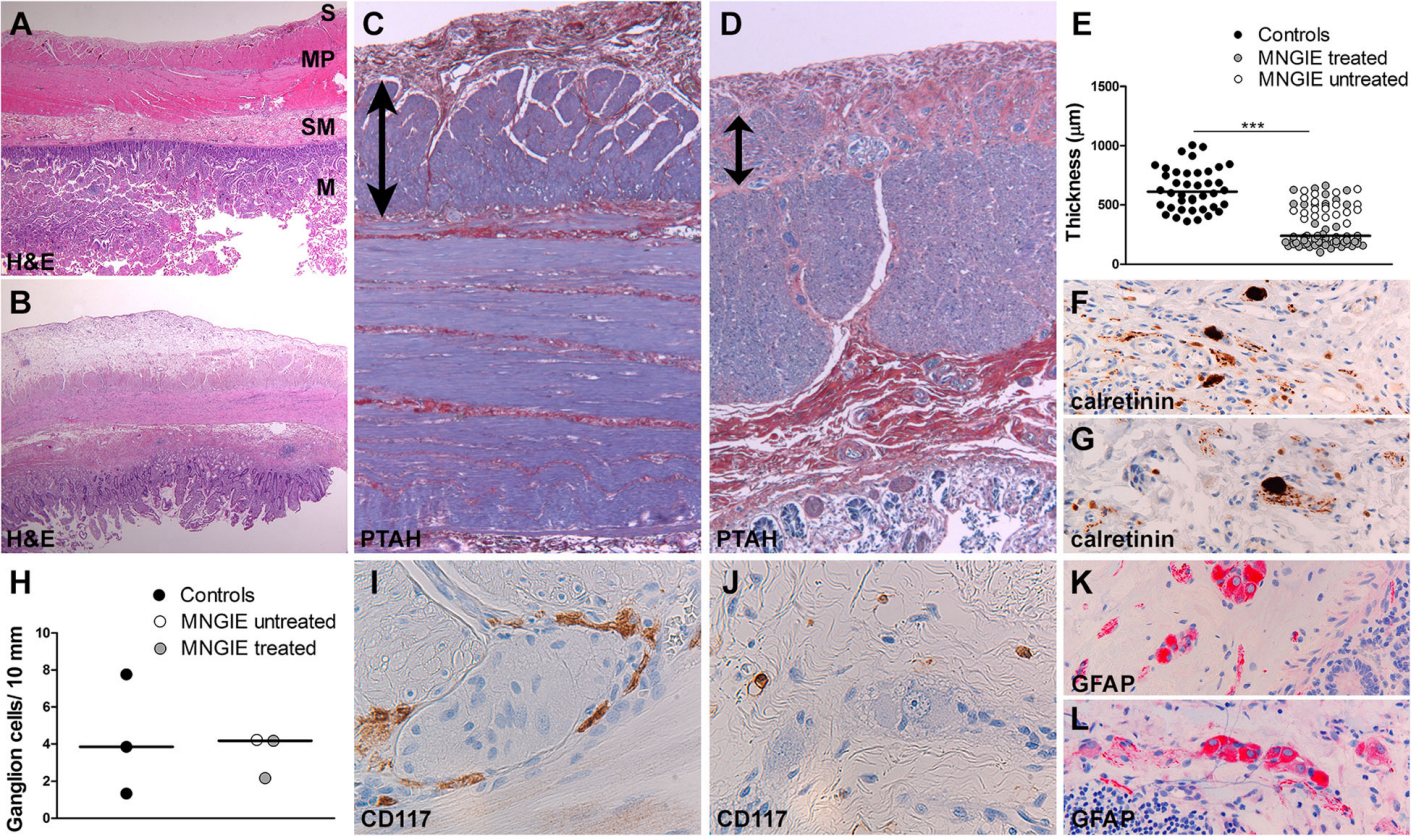
Small intestinal histopathology in MNGIE patients. **a**, **b** Hematoxylin-Eosin (H&E) stains of the small intestine of control subjects (**a**) and MNGIE patients (**b**) show normally layered organization of the wall in both groups (M: tunica mucosa; SM: tunica submucosa; MP: tunica muscularis propria; S: tunica serosa). **c**, **d** Compared to controls (**c**), phosphotungstic acid-hematoxylin (PTAH) stains of MNGIE small intestines (**d**) show thinning of the external layer of the tunica muscularis propria (blue, arrows). **e** Quantification demonstrates muscle wall atrophy in the three MNGIE patients compared to two controls. One control was omitted because the tunica muscularis was incompletely present. **f**, **g** Immunostain against calretinin shows presence of ganglion cells in the submucosal Meissner plexus of controls (**f**) and MNGIE patients (**g**). **h** Quantification of myenteric ganglion cells shows similar cell density in MNGIE patients and controls when identifying cells with calretinin (*p* = 0.99) and NeuN (*p* = 0.63, not shown). **i**, **j** Immunostain against CD117 shows normal presence of interstitial Cajal cells around grouped myenteric ganglion cells in controls (**i**), whereas Cajal cells are completely depleted in MNGIE patients (**j**). Small immunopositive cells in J are mast cells. (**k**, **l**) Immunostain against the glial fibrillary acidic protein (GFAP) shows normal immunoreactivity in myenteric ganglion and enteric glia cells in MNGIE (**l**) as in controls (**k**). In both graphs bars denote the median. Original magnifications (**a**, **b**): 12.5x; (**c**, **d**): 25x; (**i**, **j**): 400x; (**k**, **l**): 200x. ***P < 0.001

Microscopic analysis revealed a preserved layer composition of the small intestine in all MNGIE patients (Fig. 1a, b) with no villous atrophy or significant inflammation. In MNGIE patients, however, the external layer of the tunica muscularis propria was fibrotic and thinner than in controls (Fig. 1c-e), suggesting muscle atrophy. The submucosal plexus appeared normal. In the myenteric plexus, no significant loss or morphologic abnormalities of ganglion cells (identified by NeuN and calretinin immunoreactivity, Fig. 1f-h) and enteric glial cells (identified by GFAP immunoreactivity, Fig. 1k, l) were observed. Cajal cells, however, identified by CD117/c-kit immunoreactivity, were completely lost in all MNGIE patients (Fig. 1i, j).

### Small intestine pathology in MNGIE mice

**Fig. 2:**
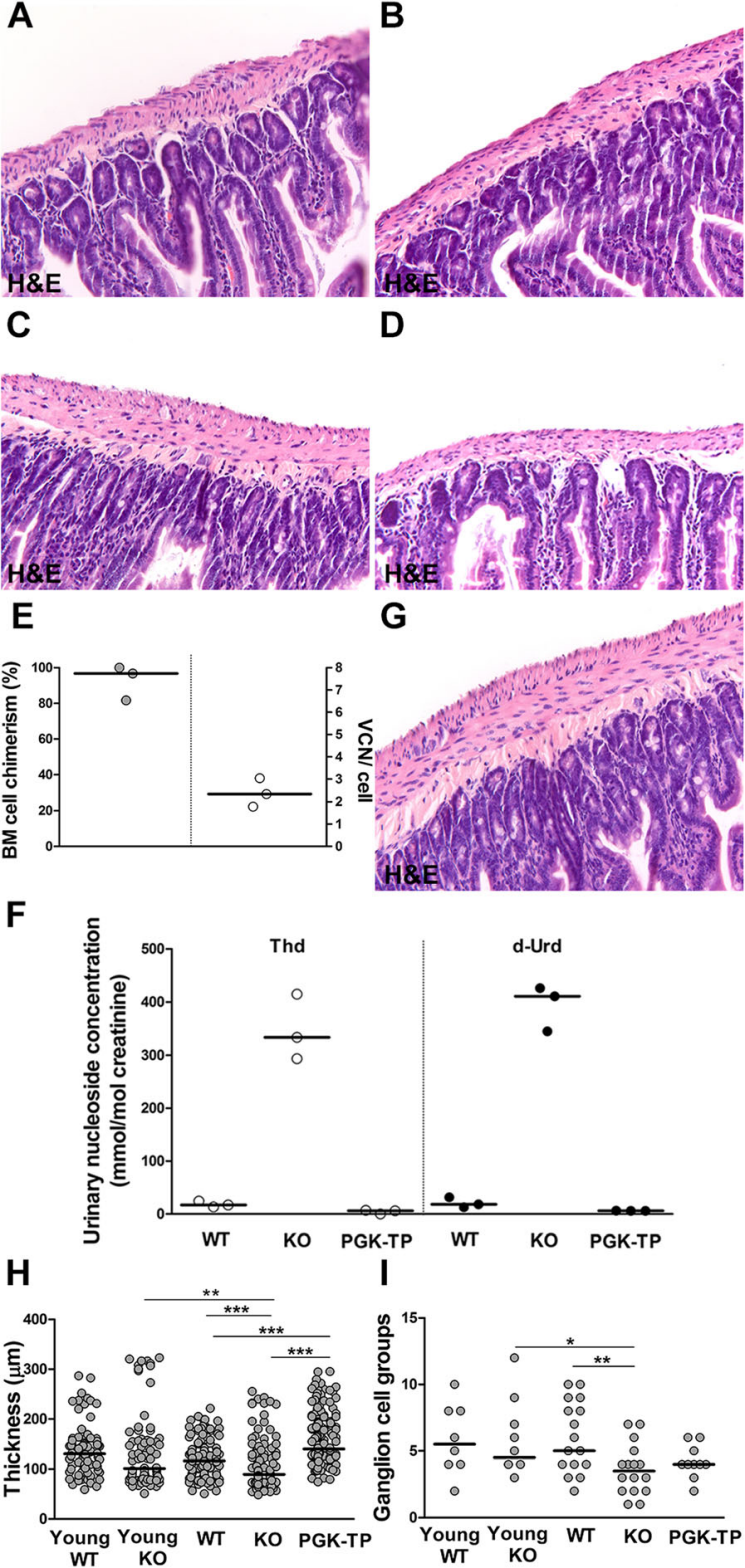
Small intestinal histopathology in MNGIE mice. **a**, **b** Hematoxylin-Eosin (H&E) stains of the small intestine of young (2-month-old) control mice (**a**) and age-matched *Tymp*^−/-^*Upp1*^−/−^ mutants (**b**) show normally layered organization of the intestinal wall in both groups. **c**, **d** H&E stains of the small intestine show normal thickness of the tunica muscularis propria in old (12-month-old) control mice (**c**), whereas in age-matched *Tymp*^−/-^*Upp1*^−/−^ mutants (**d**) the muscle wall is atrophic. **e** Bone marrow cell chimerism and vector copy number in recipients of 0.5 × 10^6^ Lin- cells transduced by LV-PGK-TP (MOI10) (*n =* 3 mice). **f** Quantification of Thd and d-Urd in urine of untreated controls and age-matched recipients 6 and 11 months after transplantation (*n =* 3 mice). **g** H&E stain of the small intestine shows that atrophy of the tunica muscularis propria is prevented in old (12-month-old) *Tymp*^−/-^*Upp1*^−/−^ mice 10 months after treatment. **h** Quantification confirms atrophy of the muscle wall in 12-month-old *Tymp*^−/-^*Upp1*^−/−^ mice compared to wild-type age-matched controls. Treatment is associated with normal thickness of the tunica muscularis propria. **i** Quantification of the number of myenteric ganglion cell groups per tissue section shows progressive loss of ganglion cells in *Tymp*^−/-^*Upp1*^−/−^ mice, without effect of the treatment. *N =* 2–4 mice/group; in all graphs lines represent the median; *P < 0.05, **P < 0.01, ***P < 0.001. Original magnification (**a**-**d** and **g**): 200x

We investigated small intestine histopathology in MNGIE mice using 2 (young) and 12-month-old animals (old) to also check for signs of progression. Hematoxylin-Eosin staining revealed significant atrophy of the tunica muscularis propria and loss of myenteric ganglion cells in old KO compared to young *Tymp*^−/-^*Upp1*^−/−^ and old WT mice (Fig. 2a-d). Unfortunately, CD117/c-kit immunostaining was unsuccessful.

We then assessed the effects of treatment on the MNGIE phenotype (Fig. 2). Following hematopoietic stem cell gene therapy (HCSGT), vector copies number per cell and engraftment levels (Fig. 2e) and urinary nucleosides concentrations (Fig. 2f) indicated efficient hematological reconstitution and biochemical correction. Histopathology showed preserved thickness of the tunica muscularis propria in old treated mice compared to *Tymp*^−/-^*Upp1*^−/−^mice (Fig. 2g, h); however, no significant changes were observed in the number of myenteric ganglion cells (Fig. 2i).

## Discussion

MNGIE is associated with gastrointestinal symptoms, including CIPO [2, 3]. Limited understanding of the pathological and molecular mechanisms underlying gastrointestinal complications in MNGIE stems from limited availability of patient tissue and models [11] that accurately recapitulate the human gastrointestinal pathology. Our study confirms the morphological changes in human MNGIE small intestine, including atrophy and fibrosis of the external layer of the tunica muscularis propria [8]. The selective involvement of this layer has been attributed to physiologically very low mitochondrial DNA amounts making this compartment selectively vulnerable to disease [8]. Muscle wall atrophy was more pronounced in transplanted patients, possibly due to additional HSCT-related stress. We also found complete loss of interstitial Cajal cells and networks in patient’s small intestine. Cajal cells play roles in orchestration of normal gastrointestinal motility and in dysmotility disorders [19]. One study previously described similar findings in a MNGIE patient [9]. Altogether, these findings suggest that Cajal cell loss is a cellular substrate of human MNGIE gastrointestinal pathology.

We used *Tymp*^−/-^*Upp1*^−/−^ mice, a model of MNGIE, to investigate whether it recapitulates the human gastrointestinal pathology. In mutant mice, histopathology revealed atrophy of the tunica muscularis propria as in MNGIE patients and, in addition, loss of myenteric ganglion cells. These features were more prominent in old *Tymp*^−/-^*Upp1*^−/−^compared to young animals. In *Tymp*^−/-^*Upp1*^−/−^ mice, we did not see presence of Cajal cell on routine Hematoxylin-Eosin stained sections. Unfortunately, CD117/c-kit immunostaining of mouse small intestine was unsuccessful, so that we cannot conclude on Cajal cells absence in *Tymp*^−/-^*Upp1*^−/−^ mice. However, taking our patient and mouse data together, we can suppose a sequence of pathological events leading to MNGIE small intestinal disease, including loss of interstitial Cajal cells and their networks followed by atrophy of the tunica muscularis propria and eventually loss of myenteric ganglion cells [9]. The observed loss of myenteric ganglion cells in the mice, but not in patient’s small intestine may be attributed to inter-species differences and the construct of the MNGIE mouse model being knock-out for both disease-related enzymes. Our study describes, for the first time, the small intestinal pathology in MNGIE patients treated with HSCT. Although the study is limited by the small patient number and short time lapse between treatment and demise, HSCT did not significantly change the trend to intestinal muscle wall atrophy and complete loss of Cajal cells in treated compared to the untreated patient. This suggests that MNGIE small intestinal pathology may not be recovered upon HSCT on the short term follow-up, which could explain the repeatedly reported insufficient improvement of gastrointestinal symptoms in MNGIE patients that do not survive short after treatment [5].

We also report for the first time that enteric glial cell morphology and density are not affected in MNGIE. Functions of enteric glial cells are currently being unraveled, and include autonomous regulation of several gastrointestinal functions, such as exocrine and endocrine secretions, motility, blood flow, and immune/inflammatory processes [20]. We were prompted to investigate glial cells in MNGIE as their central nervous system cellular counterparts, the astrocytes, are primarily affected by MNGIE and their pathology is modified by HSCGT [21].

The HSCT procedure carries a high mortality rate [5]. Recently, HSCGT has been explored in *Tymp*^−/-^*Upp1*^−/−^ mice, providing higher enzymatic levels compared to HSCT and abating the risk of graft-versus-host disease [12]. Due to intrinsic limitations of the mouse model, i.e. lack of an apparent clinical phenotype, only biochemical correction was shown after HSCGT. Moreover, the pathological changes in the intestine of *Tymp*^−/-^*Upp1*^−/−^ mice were never evaluated [12]. Here, we show that the transplanted gene modified cells engrafted well in recipient mice, leading to clearance of systemic nucleosides. The observed atrophy of the tunica muscularis propria was prevented upon HSCGT, whereas the degree of myenteric ganglion cell loss remained unchanged. Similarly as for MNGIE patients, recovery of ganglion cells may take longer than our follow-up of the mice. Alternatively, the possibility that MNGIE permanently affects ganglion cells, including their precursors, cannot be excluded.

## Conslusions

Our data suggest that allogeneic HSCT may be insufficient to correct gastrointestinal pathology completely, especially at later stages of MNGIE. As interstitial Cajal cells and their networks play a key role in development of gastrointestinal dysmotility, alternative therapeutic approaches taking absence of these cells into account could be required.

## Abbreviations

MNGIE: Mitochondrial neurogastrointestinal encephalomyopathy CIPO Chronic intestinal pseudo-obstruction HSCT Hematopoietic stem cell transplantation HCSGT Hematopoietic stem cell gene therapy

## References

[1] Hirano M, Silvestri G, Blake DM, Lombes A, Minetti C, Bonilla E, Hays AP, Lovelace RE, Butler I, Bertorini TE (1994). Mitochondrial neurogastrointestinal encephalomyopathy (MNGIE): clinical, biochemical, and genetic features of an autosomal recessive mitochondrial disorder. Neurology.

[2] Munoz MT, Solis Herruzo JA (2007). [Chronic intestinal pseudo-obstruction] Pseudo-obstruccion intestinal cronica. Rev Esp Enferm Dig.

[3] Kapur RP (2010). Pathology of intestinal motor disorders in children. Surg Pathol Clin.

[4] Yadak R, Sillevis Smitt P, van Gisbergen MW, van Til NP, de Coo IF (2017). Mitochondrial Neurogastrointestinal Encephalomyopathy caused by thymidine phosphorylase enzyme deficiency: from pathogenesis to emerging therapeutic options. Front Cell Neurosci.

[5] Halter JP, Michael W, Schupbach M, Mandel H, Casali C, Orchard K, Collin M, Valcarcel D, Rovelli A, Filosto M (2015). Allogeneic haematopoietic stem cell transplantation for mitochondrial neurogastrointestinal encephalomyopathy. Brain.

[6] Filosto M, Scarpelli M, Tonin P, Lucchini G, Pavan F, Santus F, Parini R, Donati MA, Cotelli MS, Vielmi V (2012). Course and management of allogeneic stem cell transplantation in patients with mitochondrial neurogastrointestinal encephalomyopathy. J Neurol.

[7] Perez-Atayde AR, Fox V, Teitelbaum JE, Anthony DA, Fadic R, Kalsner L, Rivkin M, Johns DR, Cox GF (1998). Mitochondrial neurogastrointestinal encephalomyopathy: diagnosis by rectal biopsy. Am J Surg Pathol.

[8] Giordano C, Sebastiani M, De Giorgio R, Travaglini C, Tancredi A, Valentino ML, Bellan M, Cossarizza A, Hirano M, d'Amati G (2008). Gastrointestinal dysmotility in mitochondrial neurogastrointestinal encephalomyopathy is caused by mitochondrial DNA depletion. Am J Pathol.

[9] Zimmer V, Feiden W, Becker G, Zimmer A, Reith W, Raedle J, Lammert F, Zeuzem S, Hirano M, Menges M (2009). Absence of the interstitial cell of Cajal network in mitochondrial neurogastrointestinal encephalomyopathy. Neurogastroenterol Motil.

[10] Perez-Atayde AR (2013). Diagnosis of mitochondrial neurogastrointestinal encephalopathy disease in gastrointestinal biopsies. Hum Pathol.

[11] Lopez LC, Akman HO, Garcia-Cazorla A, Dorado B, Marti R, Nishino I, Tadesse S, Pizzorno G, Shungu D, Bonilla E (2009). Unbalanced deoxynucleotide pools cause mitochondrial DNA instability in thymidine phosphorylase-deficient mice. Hum Mol Genet.

[12] Torres-Torronteras J, Cabrera-Perez R, Barba I, Costa C, de Luna N, Andreu AL, Barquinero J, Hirano M, Camara Y, Marti R (2016). Long-term restoration of thymidine phosphorylase function and nucleoside homeostasis using hematopoietic gene therapy in a murine model of mitochondrial Neurogastrointestinal Encephalomyopathy. Hum Gene Ther.

[13] Torres-Torronteras J, Gomez A, Eixarch H, Palenzuela L, Pizzorno G, Hirano M, Andreu AL, Barquinero J, Marti R (2011). Hematopoietic gene therapy restores thymidine phosphorylase activity in a cell culture and a murine model of MNGIE. Gene Ther.

[14] Dull T, Zufferey R, Kelly M, Mandel RJ, Nguyen M, Trono D, Naldini L (1998). A third-generation lentivirus vector with a conditional packaging system. J Virol.

[15] van Til NP, Stok M, Aerts Kaya FS, de Waard MC, Farahbakhshian E, Visser TP, Kroos MA, Jacobs EH, Willart MA, van der Wegen P (2010). Lentiviral gene therapy of murine hematopoietic stem cells ameliorates the Pompe disease phenotype. Blood.

[16] Wognum AW, Visser TP, Peters K, Bierhuizen MF, Wagemaker G (2000). Stimulation of mouse bone marrow cells with kit ligand, FLT3 ligand, and thrombopoietin leads to efficient retrovirus-mediated gene transfer to stem cells, whereas interleukin 3 and interleukin 11 reduce transduction of short- and long-term repopulating cells. Hum Gene Ther.

[17] Van Acker KJ, Eyskens FJ, Verkerk RM, Scharpe SS (1993). Urinary excretion of purine and pyrimidine metabolites in the neonate. Pediatr Res.

[18] Dubey M, Bugiani M, Ridder MC, Postma NL, Brouwers E, Polder E, Jacobs JG, Baayen JC, Klooster J, Kamermans M (2015). Mice with megalencephalic leukoencephalopathy with cysts: a developmental angle. Ann Neurol.

[19] De Giorgio R, Sarnelli G, Corinaldesi R, Stanghellini V (2004). Advances in our understanding of the pathology of chronic intestinal pseudo-obstruction. Gut.

[20] Goyal RK, Hirano I (1996). The enteric nervous system. N Engl J Med.

[21] Yadak R, Cabrera-Perez R, Torres-Torronteras J, Bugiani M, Haeck JC, Huston MW, Bogaerts E, Goffart S, Jacobs EH, Stok M (2018). Preclinical efficacy and safety evaluation of hematopoietic stem cell gene therapy in a mouse model of MNGIE. Mole Ther Methods Clin Dev.

[22] Finkenstedt A, Schranz M, Bosch S, Karall D, Burgi SS, Ensinger C, Drach M, Mayr JA, Janecke AR, Vogel W (2013). MNGIE syndrome: liver cirrhosis should be ruled out prior to bone marrow transplantation. JIMD Rep.

